# Draft Genome Sequences of Nine *Salmonella enterica* Serovar Bovismorbificans Isolates from Various Sources

**DOI:** 10.1128/genomeA.01249-13

**Published:** 2014-03-06

**Authors:** Gopal Gopinath, Junia Jean-Gilles Beaubrun, Chris Grim, Darcy Hanes

**Affiliations:** U.S. Food and Drug Administration, Center for Food Safety and Applied Nutrition, Office of Applied Research and Safety Assessment, Division of Virulence Assessment, Laurel, Maryland, USA

## Abstract

The sequences of nine genomes of *Salmonella enterica* serovar Bovismorbificans were compared to study the diversity and distribution of this emerging virulent serovar. These whole-genome sequences fill some gaps in knowledge of the diversity of the isolates used in this investigation.

## GENOME ANNOUNCEMENT

Due to the number of *Salmonella* outbreaks that have occurred in recent years, the identification, characterization, and differentiation of *Salmonella enterica* serovars involved in food-borne outbreaks are highly important concerns in microbiological analysis to aid in outbreak prevention and increase food safety. For example, *S. enterica* serovar Bovismorbificans is not commonly associated with outbreaks in the United States. The last major outbreak associated with *S*. Bovismorbificans occurred in 2004, with 35 confirmed cases associated with the consumption of alfalfa sprouts (1). From 1999 to 2009, only 758 illnesses associated with *S*. Bovismorbificans were reported, compared to 1,000 confirmed cases associated with *S. enterica* serovar Enteritidis in 1999 alone (http://www.cdc.gov/foodsafety/fdoss/data/food.html). However, in 2011, there was a multistate outbreak of *S*. Bovismorbificans infections that affected 23 people in 7 states (see http://www.cdc.gov/foodsafety/fdoss/data/food.html and http://www.cdc.gov/pulsenet/pathogens/) (2). Whole-genome sequences fill some gaps in knowledge of the diversity of these serovars. In this report, we announce the availability of nine draft genome sequences of clinical isolates of *S. enterica* serovar Bovismorbificans obtained from collections of the Center for Veterinary Medicine (SAL644), the Michigan Department of Health (SAL676, SAL677, and SAL679), the Delaware Department of Health (SAL680 and SAL681), and the Center for Food Safety and Applied Nutrition (SAL185 version 02). These genome sequences were compared to study the diversity and distribution of this emerging virulent serovar.

Genomic DNA from each strain was extracted from overnight cultures using QIAcube (Qiagen, USA). A DNA library was constructed according to the Illumina-recommended protocol for Nextera XT DNA sample prep, and the DNA was sequenced using Illumina MiSeq (San Diego, CA). CLC bio software version 6.5.1 was used for the *de novo* assembly of the trimmed paired-end reads. The resulting contigs were annotated using the RAST server (3).

The lengths and G+C contents of the draft genome sequences of the nine strains are as follows: SAL185 version 02, 4,754,300 bases and 52.2%; SAL644, 4,765,970 bases and 52.2%; SAL676, 4,569,059 bases and 52.2%; SAL677, 4,634,863 bases and 52.2%; SAL678, 4,579,778 bases and 52.1%; SAL679, 4,567,033 bases and 52.2%; SAL680, 4,596,431 bases and 52.3%; SAL681, 4,574,033 bases and 52.2%; and SAL683, 4,574,684 bases and 52.2%, respectively. Comparative analysis using RAST reveals that SAL185 and SAL644 are closely related isolates, with some phage-related proteins being exclusive to SAL185. Some of these strains have a conserved type VI secretion system. Many of these isolates form a more distinct group of *S*. Bovismorbificans strains than the 2011 Washington, DC, hummus-associated outbreak strains reported by Blaylock et al. (M. Blaylock, R. Blackwell, S. Merid, S. Jackson, M. Kotewicz, G. Gopinath, S. L. Ayers, J. Abbott, J. Sabo, L. Ewing, J. Gangiredla, S. Gebru, I. Patel, B. Jones, K. Dudley, K. Jarvis, D. E. Hanes, A. A. Diallo, and J. Jean-Gilles Beaubrun, submitted for publication) in our collection. SAL677 possesses a retron-type transposase and other markers of a transposon element in a unique 27-kb sequence among all these strains (4). Further genomic analysis of these isolates is required to understand the horizontally acquired virulence-specific genomic features in this serovar.

### Nucleotide sequence accession numbers.

The draft genome sequences for these nine *S. enterica* serovar Bovismorbificans isolates are available from the NCBI whole-genome sequence (WGS) submissions repository with the following accession no.: AZLC00000000, AZLB00000000, AZKV00000000, AZKU00000000, AZKT00000000, AZKS00000000, AZKR00000000, AZKP00000000, and AZKO00000000 (the version described here is version 02, AZKO02000000).
